# FRESH 3D Bioprinting of Collagen Types I, II, and III

**DOI:** 10.1021/acsbiomaterials.4c01826

**Published:** 2024-12-02

**Authors:** Samuel P Moss, Daniel J. Shiwarski, Adam W. Feinberg

**Affiliations:** † Department of Biomedical Engineering, Carnegie Mellon University, Pittsburgh, Pennsylvania 15213, United States of America; ‡ Department of Materials Science and Engineering, Carnegie Mellon University, Pittsburgh, Pennsylvania 15213, United States of America

**Keywords:** collagen, 3D printing, bioprinting, tissue engineering, scaffolds, FRESH

## Abstract

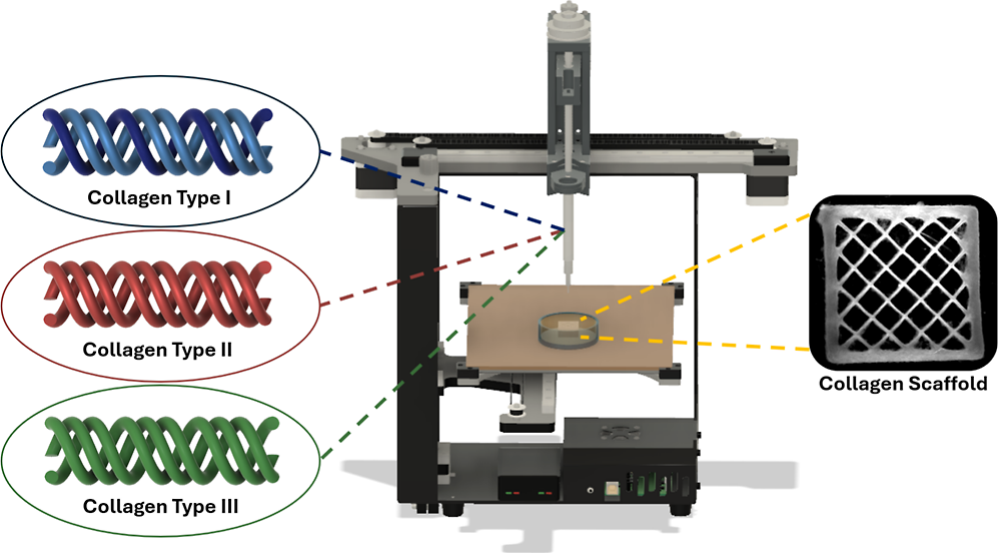

Collagens play a vital role in the mechanical integrity of tissues as well as in physical and chemical signaling throughout the body. As such, collagens are widely used biomaterials in tissue engineering; however, most 3D fabrication methods use only collagen type I and are restricted to simple cast or molded geometries that are not representative of native tissue. Freeform reversible embedding of suspended hydrogel (FRESH) 3D bioprinting has emerged as a method to fabricate complex 3D scaffolds from collagen I but has yet to be leveraged for other collagen isoforms. Here, we developed collagen type II, collagen type III, and combination bioinks for FRESH 3D bioprinting of millimeter-sized scaffolds with micrometer scale features with fidelity comparable to scaffolds fabricated with the established collagen I bioink. At the microscale, single filament extrusions were similar across all collagen bioinks with a nominal diameter of ∼100 μm using a 34-gauge needle. Scaffolds as large as 10 × 10 × 2 mm were also fabricated and showed similar overall resolution and fidelity across collagen bioinks. Finally, cell adhesion and growth on the different collagen bioinks as either cast or FRESH 3D bioprinted scaffolds were compared and found to support similar growth behaviors. In total, our results expand the range of collagen isoform bioinks that can be 3D bioprinted and demonstrate that collagen types I, II, III, and combinations thereof can all be FRESH printed with high fidelity and comparable biological response. This serves to expand the toolkit for the fabrication of tailored collagen scaffolds that can better recapitulate the extracellular matrix properties of specific tissue types.

## Introduction

As the tissue engineering field has evolved, there has been an expanding range of biomaterials and biofabrication strategies used to build tissue scaffolds. In the human body, tissues are composed of cells and the extracellular matrix (ECM), and one of the primary challenges has been using ECM proteins in their native state due to limitations in both synthesis and processing of these biopolymers. A key example of this is collagens, which are the most abundant protein type in mammals by mass and serve as the primary structural component of the ECM, containing cell and growth factor-binding sites as well as serving direct roles in tissue development and homeostasis.^1−3^ There are 28 collagen isoforms in humans, each with distinct mechanical, physical, and biochemical properties that contribute to tissue-specific ECM properties.^1^ However, specific collagen isoforms dominate in terms of mass fraction in different tissue types, often closely related to the biological structure and function. For example, collagen type I (collagen I) is the most abundant isoform overall across all tissues and organs, serving as the main source of tensile strength^4^ and making up ∼90% of the protein content in the vasculature, skin, tendon, and bone.^5^ In comparison, collagen type II (collagen II) can contain up to twice the water content than collagen I, making it more effective at dissipating compressive forces^6^ such as those found in nucleus pulposi^7^ and hyaline cartilage.^8^ Often found together with collagen type I, collagen type III (collagen III) is important for collagen I fibrillogenesis,^9^ a contributor to tissue elasticity,^10^ and is prevalent in the skin, liver, and vasculature.^11^ While there are dozens of other collagen isoforms, fibrillar collagens represent more than 90% of all collagen types.^12^ Specifically, collagens I, II, and III represent the major components of collagen fibrils^13^ and when purified and used as biomaterials are known to have excellent biocompatibility and low immunogenicity.^14^ Specific characteristics, such as molecular structure and isoelectric points of the collagen isoforms, can vary due to the purification process and species/tissue of origin.^15−17^ Further information on different collagen isoforms can be found here.^1,2,18,19^

Collagens have been widely used in tissue engineering; however, there are a limited number of fabrication techniques available, and this has been a barrier to creating more biomimetic 3D constructs. For example, collagen I is typically mammalian sourced, formed into a low viscosity solution through acidification, and then gelled via neutralization, followed by thermally induced self-assembly. The most common fabrication approaches for collagen scaffolds are electrospinning, freeze-drying, and casting techniques.^20^ Electrospinning collagen produces nanometer to micrometer fibers into nonwoven meshes that can promote cell adhesion, proliferation, and maturation;^21,22^ however, the geometry is limited to thin sheets or tubes and the small fiber spacing can limit cell migration into the scaffold. Freeze-drying collagen solutions produce porous 3D sponge-like scaffolds that cells can more readily migrate into, but 3D shapes are limited to the mold in which the freeze-drying is performed.^23−25^ Casting collagen hydrogels is the most widely used approach and produces a 3D fibrillar network that supports cell proliferation and migration but similarly is limited to simple 3D shapes or surface coatings due to the need to gel the collagen on surfaces or within molds. In general, these approaches are also limited to lower viscosity collagen I solutions of <10 mg/mL, which forms a hydrogel with an elastic modulus of ∼10 kPa^26^ or lower as compared to many tissues in the body with moduli ranging from approximately 50 to 50,000 kPa.^27^ As a result, it is often necessary to improve mechanical properties through physical or chemical cross-linking using ultraviolet light,^28,29^ γ radiation,^30^ glutaraldehyde,^31^ 1-ethyl-3-(3-(dimethylamino)propyl)-carbodiimide hydrochloride/*N*-hydroxy succinimide (EDC/NHS),^32^ or Genipin.^33^ There remains a need for biofabrication approaches capable of using multiple collagen isoforms at higher concentrations in order to achieve more biomimetic physical, mechanical, and biological properties.

The 3D bioprinting of collagen has emerged as a method to engineer tissue scaffolds that better mimic the native ECM structure and composition.^34−36^ Multiple approaches including syringe-based extrusion,^37−41^ inkjet deposition,^42−45^ and photopolymerization^46,47^ have all been demonstrated. However, the 3D complexity of these collagen-based scaffolds is still limited due to the challenges of gelling collagen as it is printed and maintaining the intended shape due to gravity-induced deformation of the soft hydrogel. This is why collagens are often mixed with other materials to achieve printable bioinks, or modified into photo-cross-linkable systems. However, these may retain cytotoxic photoinitiators and unreacted monomers or damage cells from ultraviolet-light exposure.^34^ To address these issues, extrusion-based embedded 3D bioprinting techniques deposit hydrogel bioinks within a yield-stress support bath that improves in situ gelation of collagens and physically supports the printed hydrogel in the intended geometry. Specifically, freeform reversible embedding of suspended hydrogel (FRESH) 3D bioprinting extrudes acidified collagen bioinks within a pH-buffered support bath that triggers gelation through collagen fibril self-assembly. This process has enabled the bioprinting of complex 3D scaffolds using collagen I bioink^48^ as well as decellularized ECM bioinks that contain >50% collagen I together with other ECM components.^49^ This use of decellularized ECM suggests that a broader range of collagen isoforms with different mechanical and biochemical properties could be printable.^20,50,51^

To address this capability gap, here, we demonstrate FRESH 3D bioprinting of collagen type II and collagen type III bioinks, two fibrillar collagen isoforms frequently found in human tissues. Though found in measurable quantities in many tissue types,^6−8,11^ there have been few reports trying to 3D bioprint these isoforms.^52−55^ Using the FRESH 3D bioprinting platform, we assessed single printed filaments of each bioink and verified the similar microstructure between filaments of all three collagen isoform bioinks. We then produced centimeter-scale scaffolds with micrometer-scale features with high fidelity and reproducibility, confirming that the collagen II and collagen III bioinks have a printability on par with the established collagen I bioink. Finally, we confirmed that cell viability and proliferation on all collagen scaffolds (I, II, and III) were comparable. Altogether, we have demonstrated the ability to fabricate scaffolds using the three most common collagen isoforms alone or in combination, thus expanding the range of ECM compositions that can be printed to better mimic the mechanical and biochemical properties of different tissue types.

## Experimental Section (Materials and Methods)

### FRESH Support Bath Preparation

The FRESH gelatin microparticle support bath was prepared using a complex coacervation based on previously described methods.^48^ Briefly, 2.0% (w/v) gelatin type B (Rousselot), 0.25% (w/v) Pluronic F-127 (Sigma-Aldrich), and 0.5% (w/v) gum arabic (Sigma-Aldrich) were dissolved in 50% (v/v) 200 proof ethanol in distilled water at 45 °C in a 1 L beaker. The pH was adjusted to 5.82 by the addition of 1 M hydrochloric acid. Note that the pH to which the solution is adjusted for coacervation will vary depending on the isoelectric point of the batch of gelatin used in this process. An overhead stirrer (IKA, Model RW20) was used to stir the solution overnight at 560 rpm. To remove ethanol and Pluronic F-127 from the gelatin microparticles, a series of washing steps was performed with distilled water followed by washing steps with aqueous 4-(2-hydroxyethyl)-1-piperazineethanesulfonic acid (HEPES) solutions. The rotor was turned off at least 1 h prior to washing steps to allow the gelatin microparticles to settle in solution. The supernatant was decanted before the remaining solution was placed in 250 mL polycarbonate Nalgene containers and centrifuged at 850 rcf for 4 min. The supernatant was decanted, and the containers were filled with distilled water and then vortexed for 30 s. This step was repeated once more with distilled water and then two more times with aqueous HEPES solutions. The final centrifugation was performed at 2000 rcf to compact the microparticles together to serve as the support bath. The compacted gelatin microparticles were then transferred into 35 mm Petri dishes (Corning) for printing. The support bath was washed with 50 mM HEPES at pH = 7.4 for collagen I bioink. For scaffolds printed with the collagen II, III, and combinatorial bioinks, the support bath was washed with 100 mM HEPES at pH = 7.4 to increase buffering due to the greater acidity of these bioinks.

### Preparation of Collagen Bioinks

The collagen II (Sigma-Aldrich C9301) and III (Advanced Biomatrix 5019) powders were dissolved in 0.24 M acetic acid at 4 °C to a concentration of 20 mg/mL in a 1 mL syringe (Becton Dickinson). This was then placed on a rocker plate for 48 h at 4 °C until the powders were completely dissolved. The 1 mL syringe was then centrifuged in a custom adapter for 10 min at 3000 rcf to remove air bubbles. The collagen I bioink was prepared by acidifying the neutral LifeInk 200 with 0.24 M acetic acid with 1-part acetic acid to 2-parts LifeInk 200. The bioink was then diluted with 0.08 M acetic acid from 23 to 20 mg/mL to match the concentration of the collagen II and III bioinks. A higher concentration of 0.24 M acetic acid was used to dissolve collagen II and III in their respective bioinks because the powders did not completely dissolve at the 0.08 M acetic acid concentration. After centrifugation, bioinks were loaded into their own 500 μL gastight glass syringe (Hamilton 81222) for printing.

### FRESH 3D Bioprinting

Single filaments and square lattice designs were FRESH printed based on previously described methods.^48^ Briefly, the 3D models were generated by using Fusion 360 (Autodesk) computer-aided design software. The single suspended filaments were placed within a window frame support, consisting of a 9 × 4 × 1 mm rectangular solid with five 1 × 2 mm holes in which a single 0.15 mm wide, 2 mm long, and 0.06 mm thick filament was suspended. For the square lattice, a 10 mm × 10 mm × 2 mm rectangular solid with 20% infill was used. For cell seeding experiments, a circular disk with a diameter of 8 mm, a height of 0.48 mm, and 100% infill was used. Layer heights were set at approximately 40% of the needle’s inner diameter used for printing. All prints used a 30G needle with a layer height of 60 μm, unless otherwise specified. Cura 4.13.1 (Ultimaker) slicer software was used to process the 3D models into G-codes using print parameters appropriate to the needle and syringe diameters being used. These G-codes were loaded onto a custom-built bioprinter based on a MakerBot Replicator 2× with Replistruder 4 syringe pumps^56,57^ loaded with syringes for the different bioinks. FRESH printing was then performed by extruding the bioinks within the gelatin microparticle support bath.^48^ After printing, the collagen scaffolds were released from the FRESH support bath by placing the print containers in an incubator at 37 °C and exchanging the liquified gelatin with warm 1× PBS. This was performed 3 times with at least 45 min of incubation between each exchange to ensure complete melting and removal of any remaining gelatin microparticles within the scaffolds.

### Casted Collagen Hydrogel Formation

Casted collagen hydrogels were fabricated by adapting the manufacturer’s Corning Collagen I High Concentration, Rat Tail alternate gelation procedure.^58^ A total volume of 300 μL for the collagen I condition was prepared consisting of 30 μL of 10× PBS, 7.2 μL of 1 N NaOH, 172.8 μL of distilled water, and 90 μL of acidified LifeInk 240 at 20 mg/mL combined together in this order. The same steps were followed for the collagen II and III conditions, with slightly different volumes of 30 μL of 10× PBS, 20.7 μL of NaOH, 159.3 μL of distilled water, and 90 μL of the already acidified collagen bioink. Collagen discs were molded by casting 45 μL of the solution into circular molds with 8 mm diameters made from silicone gaskets on sonicated coverslips and then placed in an incubator for 3 h to allow for thermal gelation.

### Cell Culture, Seeding, and Staining

To expand cells for these studies, murine C2C12 myoblasts (ATCC CRL-1772) were cultured at 37 °C under 10% CO_2_ with Dulbecco’s modified Eagle’s medium (Corning) supplemented with 10% (v/v) Fetal Bovine Serum (VWR), 1% (v/v) l-glutamine (Life Technologies), and 1% (v/v) penicillin–streptomycin (Life Technologies). The medium was exchanged every 2 days and C2C12s were passaged prior to reaching 80% confluence. Prior to cell seeding, both casted and FRESH printed collagen scaffolds were placed in a biological safety cabinet to dry for ∼12 h and then rehydrated by submerging them in 1× PBS. The scaffolds were then sterilized by placing them in a Novascan PSD Pro-UV6 which emits ultraviolet light at an extrapolated irradiance of 28–32 mW/cm^2^ at 253.7 nm with a distance of 10 cm for 15 min. The exposure to ultraviolet light may cause further cross-linking. This was followed by seeding with 500000 C2C12 mouse myoblast cells per sample in 6-well plates. The seeded scaffolds were placed in the incubator for 1 h and 30 min before adding an additional 2 mL of C2C12 growth media to the well. Seeded scaffolds were rinsed with 1× dPBS 24 and 48 h after seeding and replenished with 2 mL of C2C12 growth media. After 3 days in culture, collagen scaffolds seeded with C2C12 cells were fixed using 4% paraformaldehyde with 0.05% Triton-X for 20 min. After fixing, the gels were washed 3× with 1× PBS and a 10 min period between each PBS wash. Cells were then stained with To-Pro-3 (Thermo Fisher T3605) and Alexa Fluor 488 Phalloidin (Thermo Fisher A12379) according to the manufacturer’s instructions.

### Optical Imaging of Collagen Scaffolds

Multiple optical image techniques were used to assess the collagen scaffolds and seeded cells. The single collagen filaments were imaged with a 10× or 16× objective using 488 nm reflectance confocal microscopy (A1R Nikon) while submerged in 1× PBS. The 3D images of the filaments were rendered in Imaris 9.1 (Bitplane) using the normal shading tool. Diameters of each filament were determined by taking the average of 5 measurements at different locations of the filament in the direction perpendicular to filament direction in ImageJ (National Institutes of Health) for broad characterization across their entire length. The collagen square lattice scaffolds were imaged under darkfield with a stereomicroscope (M165 FC Leica) using a Prime 95B Scientific CMOS camera (Photometrics) while submerged in PBS. The fluorescently labeled collagen scaffolds with cells were imaged using laser scanning confocal microscopy (A1R Nikon) and the unlabeled collagen was imaged using multiphoton microscopy (A1R Nikon) via second harmonic imaging.

## Results

### 3D Bioprinting of Collagen Type I, II, and III Filaments

To compare across collagen bioink types and assess basic printability, we FRESH printed single filaments and analyzed the microstructure using reflectance confocal microscopy. We used the same printing process previously reported for collagen I and decellularized ECM,^48^ which is extrusion of the bioink as an acidified collagen solution into a HEPES-buffered gelatin microparticle support bath. This rapidly neutralizes the pH driving collagen gelation through fibrillogenesis, creating physical cross-linking into a 3D fibrillar network. The FRESH-printed collagen then undergoes further thermally driven physical cross-linking as the support bath is melted in an incubator at 37 °C to release the printed part. To determine if this approach could be applied to collagens II and III, we created our own bioinks by dissolving these collagens at a concentration of 20 mg/mL in water and acetic acid, matching the formulation of our standard collagen I bioink.

**Figure 1:**
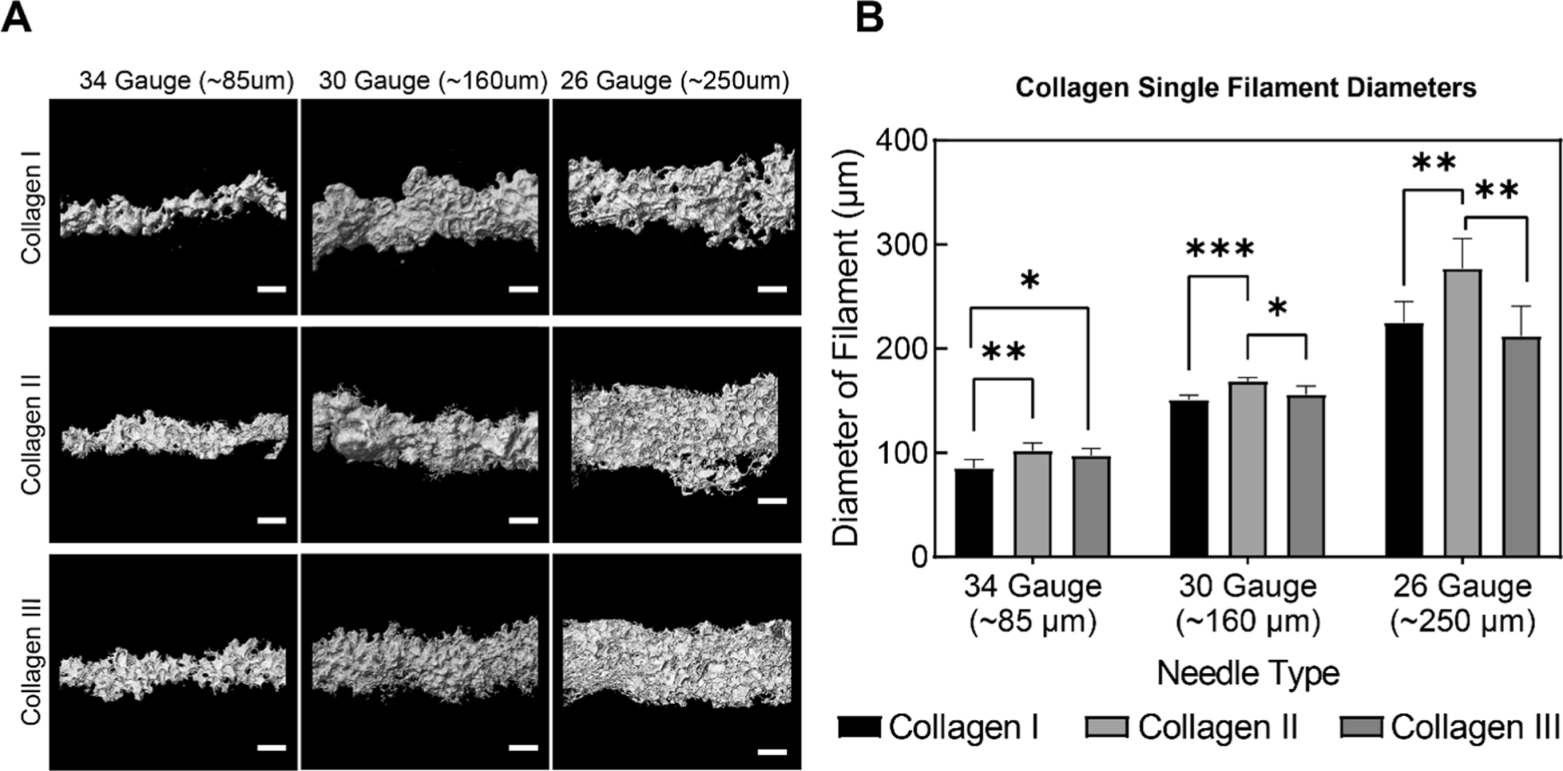
Collagen isoform bioinks yield filaments with similar microstructure topography. (A) Reflectance confocal microscopy images of FRESH 3D bioprinted single filaments of collagen I, II, and III bioinks extruded from 34-, 30-, and 26-gauge needles. Scale bars are 100 μm. (B) Measured mean diameters of collagen filaments, which can be compared to the nominal inner diameter of the needles used for extrusion (*n* = 5, ±SD, * indicates *p* < 0.05, ** indicates *p* < 0.01, *** indicates *p* < 0.001, statistical analysis is one-way ANOVA with Tukey’s multiple pairwise comparisons).

Overall, results show that the collagen I, II, and III bioinks were printed in a similar fashion to each other, with only minor differences. Filaments of collagen I, II, and III were FRESH printed with 26-, 30-, and 34-gauge needles, corresponding to nozzle inner diameters of approximately 250, 160, and 85 μm, respectively (Figure 1A). The smallest 34-gauge needle with a nominal 85 μm inner diameter produced filaments of a comparable size, though the collagen II and III filaments were slightly larger in diameter than collagen I (Figure 1B). Similar results were obtained for the 30- and 26-gauge needle conditions (Figure 1A), though collagen II showed a significant increase in the filament diameter compared to collagens I and III with an overall increase in variability of all filaments printed with the 26-gauge needles (Figure 1B). Across all needle sizes and collagen types, it was clear that the microparticles in the support bath influenced the filament morphology, primarily causing a dimpled appearance on the filament surface. This is a characteristic of FRESH printing that we have shown in previous work and can be reduced or entirely eliminated by changing the pH and/or salt concentration of the support bath.^48^ While this might be viewed as a negative, we have found that when multiple filaments are printed together into a larger part, this morphology improves filament-to-filament adhesion and provides a surface that promotes cell attachment. From a print fidelity standpoint, the slightly larger diameter of collagen II filaments means that a correction factor may need to be applied when slicing 3D models into the G-code in order to account for this volumetric difference.

### 3D Bioprinting of Collagen Type I, II, and III Scaffolds

**Figure 2:**
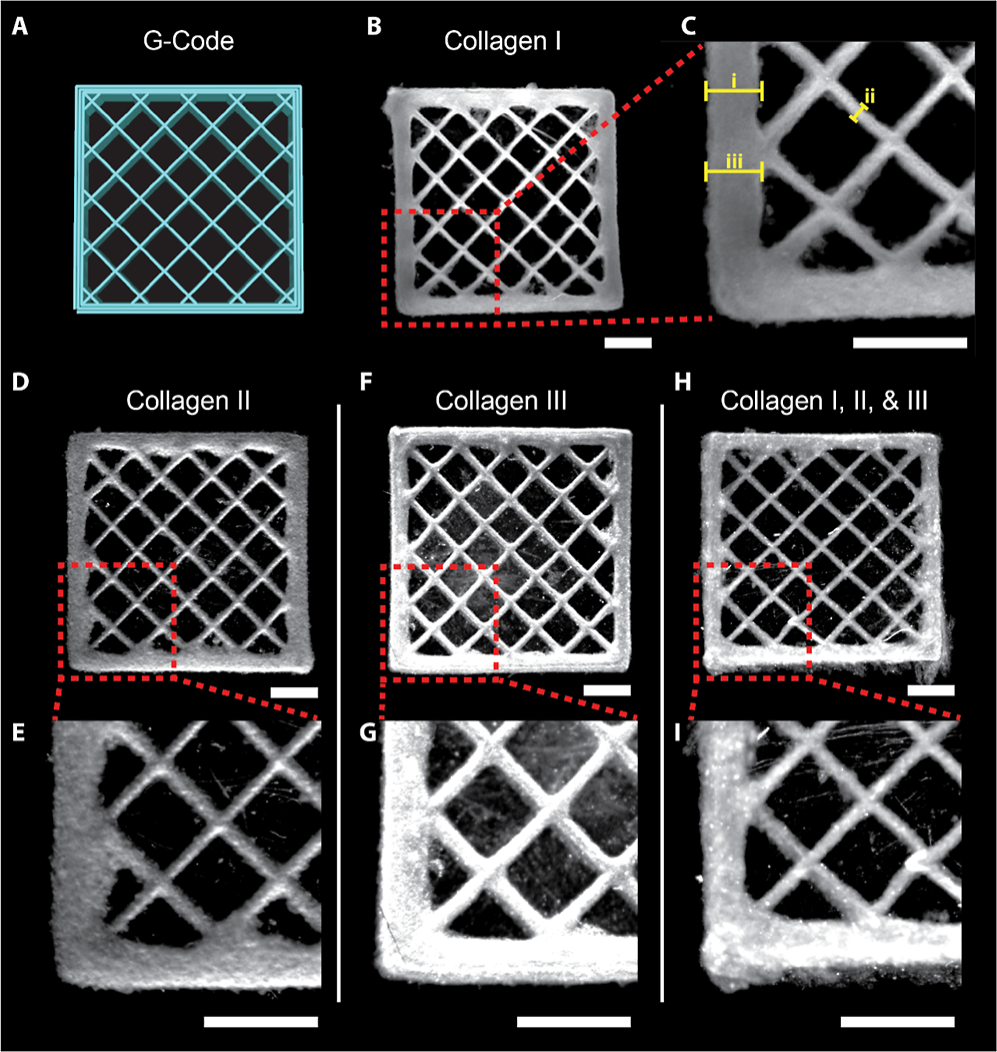
Macroscale collagen scaffold fabrication displays similar printing characteristics between collagen isoform bioinks. (A) G-Code depicting the print pathing of a 3D grid with a square cross-section of 10 mm × 10 mm and 2 mm in height. (B,C) Stereo microscope images of the bioprinted collagen I scaffold and a zoomed-in ROI showing the fine detail of the corner of the printed scaffold. (D,E) Stereo microscope images of the bioprinted collagen II scaffold and a zoomed-in ROI showing the fine detail of the corner of the printed scaffold. (F,G) Stereo microscope images of the bioprinted collagen III scaffold and a zoomed-in ROI showing the fine detail of the corner of the printed scaffold. (H,I) Stereo microscope images of the bioprinted collagen I, II, and III scaffold and a zoomed-in ROI showing the fine detail of the corner of the printed scaffold. All scale bars are 2000 μm.

Next, we assessed the ability to FRESH print more complex 3D scaffolds from collagens I, II, and III and a combination of all three. To do this, we printed a 10 mm square with a rectilinear grid infill in order to assess filament fusion and overall fidelity (Figure 2A). The collagen I bioink was used as a reference for print quality between conditions and as expected accurately recreated the intended geometry with high fidelity (Figure 2B). A further region of interest (ROI) (Figure 2C) was defined to highlight fine features: the wall (Figure 2Ci), infill (Figure 2Cii), and intersection of these two features (Figure 2Ciii). The collagen II bioink accurately recreated the intended grid geometry (Figure 2D); however, some of the finer features were slightly deformed in comparison to those of collagen I (Figure 2E). The infill was consistent with the infill depicted in the collagen I scaffold, but the wall feature was slightly deformed. Specifically, the point where the wall and infill intersect was statistically larger in the collagen II scaffold than in collagen I (Figure S1). The wall at the intersection was also statistically larger than the wall not at the intersection in the collagen II scaffold. The collagen III bioink printed on par with the collagen I bioink (Figure 2F) and all three features had dimensions in the collagen III scaffold (Figure 2G) comparable to those of the collagen I scaffold. We also explored the ability to use these collagen isoforms together by creating a combinatorial bioink consisting of equal parts of collagen I, II, and III bioinks. The combinatorial bioink also exhibited characteristics similar to those of the collagen I bioink scaffold (Figure 2H) with all three finer features being of similar quality (Figure 2I). Quantitative measurements for all square lattice scaffolds can be found in Figure S1. This demonstrates the feasibility of combining the fibrillar collagen isoform bioinks to enable the creation of custom formulations depending on the application.

#### Cellular Response to Different Collagen Bioinks

**Figure 3:**
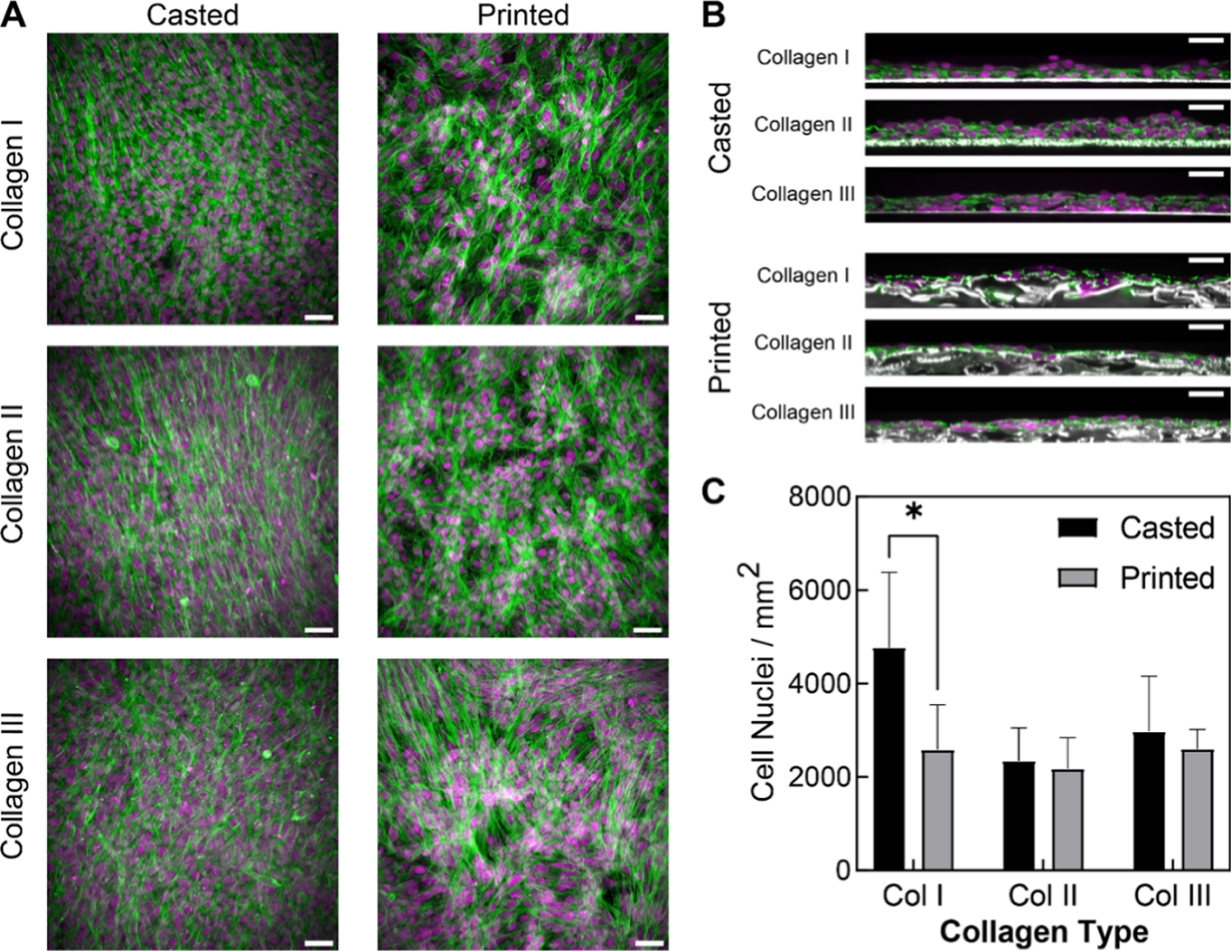
Similar cellular responses on scaffolds from all collagen isoform bioinks in both casted and FRESH-printed conditions despite the change in topology. (A) C2C12 mouse myoblasts adhered and spread across printed and casted collagen gels, stained for f-actin (green) and nuclei (magenta) show cell attachment and spreading. (B) Multiphoton images of C2C12s on the collagen gels in the xz orientation depicting the dimpled topology of the printed conditions with f-actin (green), nuclei (magenta), and collagen (white). (C) Quantification of cell nuclei density for all bioink and fabrication conditions (*n* ≥ 4, ±SD, *p < 0.05, two-way ANOVA with Tukey’s multiple comparison tests). All scale bars are 50 μm.

To understand whether collagen isoform impacts cell attachment and growth, we seeded FRESH-printed collagen scaffolds with C2C12 mouse myoblasts and compared them to casted controls. All of the collagen bioinks supported the attachment and spreading of C2C12 mouse myoblasts on both cast and printed scaffolds (Figure 3A). There was no observable distinction between the collagen types, with each condition showing complete cell coverage across the scaffold surface after 3 days of culture. The images were mostly devoid of balled up or detached cells, suggesting that the collagen scaffolds provided a surface that allowed for the attachment and proliferation of the cells. There was a notable qualitative difference in cell morphology between the casted and printed conditions, with the latter having more variability in the actin cytoskeleton (Figure 3A). This was due to a difference in surface topology between the casted and printed collagen scaffolds, with the latter showing some surface undulations in the cross-section (Figure 3B). Despite these differences in morphology, cell density showed a significant difference between the casted and printed condition for collagen I, but cell density for all other conditions was statistically equivalent (Figure 3C). The reason for higher cell density on casted collagen I is not entirely clear, though in part, it may be due to initially faster cell attachment and proliferation. We have previously observed the faster cell attachment to smooth collagen gels than collagen gels with a dimpled surface; however, we have not characterized the cause of this at this time. It is possible that with a longer incubation period, all conditions would exhibit similar cell coverage numbers, but this would need to be verified through future studies. Altogether, this data establishes that the collagen scaffolds fabricated with these bioinks produce scaffolds capable of supporting cell attachment and proliferation.

## Discussion

While all collagen bioinks performed similarly, the slight differences in filament sizes highlight the importance of collagen gelation during the printing process. Slightly thicker filaments were observed in all collagen II conditions and one collagen III condition, which may be due to the bioinks having a higher acidity than the collagen I bioink. The higher acidity was required to prevent gelation of collagen II and III as the respective powders were dissolved in acetic acid. This higher acidity may have caused the bioinks to take longer to neutralize in the HEPES-buffered support bath, which would allow the ink to diffuse further throughout the bath before gelling. This would indicate that the filament characteristics could be controlled through the rate of neutralization with factors such as the bioink acidity or buffering concentration of the support bath. While all bioinks have qualitatively similar viscosities, small differences between each could be another cause for filament diameter variations. Lower viscosity bioinks should be able to diffuse further into the support bath before undergoing gelation, leading to larger filament dimensions. Differences in swelling ratios between each hydrogel are an additional cause that could lead to the variation. All collagen hydrogels display very similar characteristics; therefore, we do not believe this to be the driving factor in filament size variation. Another potential cause for this behavior is that each collagen isoform has a different number of titratable groups which play a direct role in the pH of a collagen solution and therefore should alter the gelation rate between isoforms.^59^ The extraction and processing methods of each collagen type can also play a role in affecting gelation rates as they may slightly alter the proteins and remove side groups important in determining pH such as carboxylic acids.^5^ Any of these factors or a combination of them may have caused the larger filament size observed with the collagen II bioink. Specifically, the larger filaments printed with a 30-gauge needle may explain the behavior found in Figure 2E where the intersection of the wall and infill was enlarged. The larger filament size will have created a larger overlap between the infill and wall filaments, causing the features to blend together, leading to thicker features at this intersection.

The establishment of collagen II and III bioinks enables the biofabrication of collagen scaffolds with the ability to tailor mechanical and biological properties. In general, collagen is widely used throughout tissue engineering due to its natural role in the ECM as the main structural component, multiple cell and growth factor-binding domains, and as a consequence of its role in biomechanical and biochemical signaling.^20,60^ Currently, collagen I scaffolds have been widely used in cartilage tissue engineering applications; however, collagen II has shown better cartilage regeneration and chondrogenic induction and maintenance.^50,51,61,62^ In these published studies, the molding and lyophilization techniques used to fabricate these scaffolds limit the possible geometries. With the results reported here, the collagen II bioink could be readily adapted for cartilage tissue engineering applications with the ability to fabricate a range of physiologically relevant scaffold geometries with FRESH 3D bioprinting. In terms of collagen III, it is typically found together with collagen I in the body, suggesting that the collagen III bioink may be best in a blended formulation with collagen I. Collagen III has been used in limited quantities in tissue engineering and is most commonly viewed as a contaminant in collagen I derived from the skin.^20^ Collagen III contributes to the elasticity of the ECM,^10^ which coincides with it being found in higher quantities, up to 40% of the collagen content, in more elastic tissues such as blood vessels, vocal folds, lung, intestine, liver, and skin.^11,63^ Additionally, collagen III has been shown to play a critical role in both in vivo and in vitro fibrillogenesis of collagen I.^9,64^ This data suggests that the collagen III bioink could be combined with collagen I specifically to achieve tunability of mechanical properties of the collagen I/III scaffolds to match the intended target tissue.

The pH-induced fibrillogenesis process of these bioinks during FRESH 3D bioprinting suggests that this biofabrication approach is highly adaptable and could be used for an even broader range of collagen types and tissue engineering applications. The robustness of this approach should be noted as all three bioinks are sourced from different tissue and species with collagen I, II, and III coming from the bovine skin, chicken sternum, and human placenta, respectively. This suggests that fibrillar collagen types, regardless of tissue or species of origin, can be adapted to this approach. Though not tested and difficult to obtain in purified form commercially, it is thus probable that the FRESH printing of acidified collagen bioinks and subsequent neutralization process could be adapted to other fibrillar collagen types such as V, XI, XXIV, and XXVII.^65^ Of course, collagens only make up ∼30% of the ECM in most tissues,^3^ and we have previously reported that purified collagen bioinks can be combined with decellularized ECM and FRESH 3D bioprinted to form tissue-specific scaffold composition and 3D structure.^49^ Further, these collagen isoform bioinks can also be used in concert with other bioinks that have orthogonal gelation and cross-linking mechanisms that also work in FRESH 3D bioprinting, such as fibrinogen, alginate, and photo-cross-linkable hydrogels.^48,66^

The ability to use these collagen bioinks together with other types of bioinks is important in order to engineer more complex and cellularized tissue constructs. Specifically, the acidity of the collagen bioinks means that we cannot mix cells with the collagen solution because the low pH would kill them. Since the acidic pH is required for the rapid neutralization of the collagen bioink when exposed to the pH-buffered support bath, which initiates collagen fibrillogenesis, we cannot simply neutralize the collagen prior to printing.^67^ Alternatively, a neutral pH collagen bioink could incorporate cells, but it is challenging to achieve this without gelling the bioink prematurely within the syringe. The higher viscosity of a fully or partially gelled bioink will increase the shear stress on cells as they are extruded, potentially leading to decreased cell viability.^68^ Neutral pH cell-laden collagen bioinks that have been reported rely on post printing thermal gelation to fuse printed filaments together.^69^ However, these approaches have notably worse print fidelity in large part due to reduced filament-to-filament adhesion, which impacts overall scaffold integrity. Instead, pH-neutral cell-laden bioinks such as fibrinogen or alginate are capable of being used to print cells in FRESH 3D bioprinting, and in multimaterial designs, they can be coprinted with acidified collagen bioinks to achieve fully integrated and cellularized tissue constructs.^48,70,71^

## Conclusions

In summary, FRESH 3D bioprinting has demonstrated the capability to use collagen I, collagen II, and collagen III bioinks and combinations thereof with comparable print fidelity. There were only some small variations in filament diameter, likely due to slight differences in bioink formulation. The use of collagen bioinks with a range of isoforms is important because it enables the fabrication of scaffolds that better match the ECM composition of cartilage, vasculature, skin, and other tissue types. The relatively straightforward success of FRESH printing collagen II and III bioinks using the same basic process as for collagen I bioink suggests the ability to FRESH print other fibrillar collagens. The reason we did not investigate these other fibrillar collagens in this current work is the challenge of obtaining sufficient quantities from commercial sources. However, it is possible to produce many of the other fibrillar collagen isoforms recombinantly, providing a future pathway to expand the range of bioinks that can be FRESH printed. Overall, the ability to use pH-triggered gelation of multiple fibrillar collagen bioinks together with other bioinks using orthogonal enzymatic, ionic, and light-based gelation mechanisms enables the FRESH 3D bioprinting of multimaterial cell-laden scaffolds that better matches the structure and composition of native tissues.

## Supporting Information Available

Quantification of print features (PDF)
