# Supplementary material for: FRESH 3D Bioprinting of Collagen Types I, II, and III
Source: ACS Biomater Sci Eng. 2024 Dec 2;11(1):556–63. doi: 10.1021/acsbiomaterials.4c01826 (PMC11733922; doi:10.1021/acsbiomaterials.4c01826)
Supplement: Supplementary file 1 — ab4c01826_si_001.pdf [file ab4c01826_si_001.pdf]

Supporting Information

FRESH 3D Bioprinting of Collagen Types I, II, and III

Samuel P. Moss [1], Daniel J. Shiwarski [1], and Adam W. Feinberg [1,2].*

[1] Department of Biomedical Engineering, Carnegie Mellon University, Pittsburgh, United States of America.

[2] Department of Materials Science and Engineering, Carnegie Mellon University, Pittsburgh, United States of America.

* Correspondence: feinberg@andrew.cmu.edu

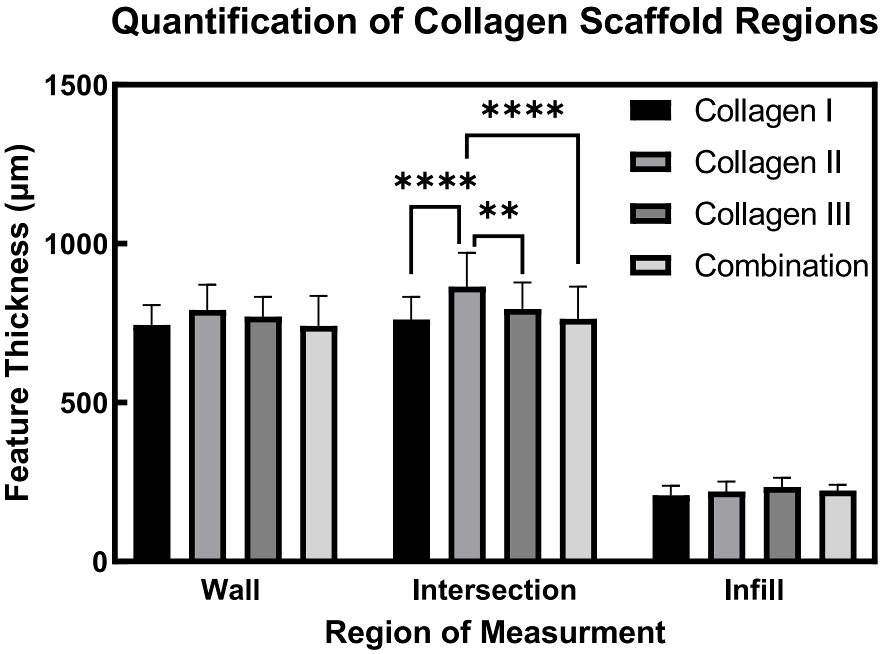

Figure S1. Quantification of print features in collagen type I, II, and III scaffolds. Measurements of print features in scaffolds of all three collagen types and an equal combination of each. The measurements for the wall are collagen I (744 ± 63 μm), II (791 ± 79 μm), III (770 ± 63 μm), and combination (742 ± 94 μm). The measurements for the intersection are collagen I (761 ± 71 μm), II (865 ± 107 μm), III (794 ± 84 μm), and combination (764 ± 101 μm). The measurements for the infill are collagen I (209 ± 30 μm), II (221 ± 31 μm), III (, 235 ± 29 μm), and combination (224 ± 17 μm). (wall n = 16, intersection and infill n =32, ± SD, ** indicates $p<0.01$, **** indicates $p<0.0001$, statistical analysis is a one-way ANOVA with Tukey's multiple pairwise comparisons)
